# Physical Interaction of Sodium Houttuyfonate With β-1,3-Glucan Evokes *Candida albicans* Cell Wall Remodeling

**DOI:** 10.3389/fmicb.2019.00034

**Published:** 2019-01-25

**Authors:** Wenyue Da, Jing Shao, Qianqian Li, Gaoxiang Shi, Tianming Wang, Daqiang Wu, Changzhong Wang

**Affiliations:** Department of Pathogenic Biology and Immunology, College of Integrated Chinese and Western Medicine (College of Life Science), Anhui University of Chinese Medicine, Hefei, China

**Keywords:** *Candida albicans*, sodium houttuyfonate, glucan, chitin, unmasking, cell wall remodeling

## Abstract

*Candida albicans* is a commonly isolated opportunistic yeast and can endanger immune-compromised human health. As increasingly isolated strains present resistance to currently used antifungals, it is necessary to develop novel antimycotics. In a previous study, sodium houttuyfonate (SH) alone or in combination with fluconazole revealed relatively strong antifungal potential against *C. albicans*, and the underlying mechanism might be likely to be associated with β-glucan synthesis and transportation (Shao et al., 2017). In the present experiment, we used a standard *C. albicans* isolate and a phr1 mutant (*phr1*−/−) to investigate the interaction of SH with β-glucan, one of the critical components in cell wall and biofilm matrix. We showed that lyticase was the most effective enzyme that could significantly increase the antifungal inhibition of SH at 64 μg/mL in *C. albicans* SC5314 but became futile in *phr1*−/−. Although the minimum inhibitory concentrations (MICs) of SH were comparable in the two *Candida* strains used, *phr1*−/− appeared to be more susceptible to SH compared with *C. albicans* SC5314 in biofilms (64 versus 512 μg/mL). The peak areas of SH decreased markedly by 71.6, 38.2, and 62.6% in *C. albicans* SC5314 and by 70% and 53.2% in *phr1*−/− by ultra-performance liquid chromatography (UPLC) analysis after co-incubation of SH with laminarin, extracellular matrix (EM) and cell wall. The chitin appeared to not interact with SH. We further demonstrated that sub-MIC SH (8 μg/mL) was able to induce cell wall remodeling by unmasking β-1,3-glucan and chitin in both *C. albicans* SC5314 and *phr1*−/−. Based on these findings, we propose that β-1,3-glucan can block the entrance of SH through non-specific absorption, and then the fungus senses the interaction of SH with β-1,3-glucan and exposes more β-1,3-glucan that contributes to SH blocking in turn.

## Introduction

*Candida albicans*, a frequently isolated opportunistic pathogen, presents with two opposite faces, i.e., an ordinary resident on the skin and mucosal layers of healthy people, and a fatal invader causing deep-seated systematic infections in compromised individuals (Poulain, 2013). *C. albicans* possesses an arsenal of effective tools to overcome extraneous scavengers (especially antifungal drugs). These tools include the secretions of invasive enzyme (such as secreted aspartyl proteinases) and quorum sensing molecules (mainly referring to farnesol and tyrosol), the ability of yeast-to-hypha transition and biofilm formation, the working of efflux pumps (such as *cdr1*, *cdr2*, and *mdr1*), and the mutations of key genes that are the targets of antifungal agents (such as *erg11* and *fks1* mutants) (Hornby et al., 2001; Chen et al., 2004; Flemming and Wingender, 2010; Shapiro et al., 2011; Sudbery, 2011; Fan et al., 2013; Taff et al., 2013; Prasad and Rawal, 2014; Robbins et al., 2017). Since there are multiple factors influencing *C. albicans* resistance, it becomes necessary to develop novel drugs as synergists to improve the antifungal effects of conventional agents.

It is economic and time-saving to exploit drugs with antifungal potentials which usually have anti-inflammatory, anti-tumor, anti-virus, and anti-bacterial functions (Liu et al., 2011; Lu et al., 2017). Sodium houttuyfonate [SH, CH_3_(CH_2_)_8_COCH_2_CHOHSO_3_Na], a derivative of houttuynin [CH_3_(CH_2_)_8_COCH_2_CHO], is one of the main and most effective compounds extracted from *Houttuynia cordata* Thunb (Saururaceae family) (Shao et al., 2012). SH was not only effective against gram positive bacteria (such as *Staphylococcus aureus* and *Bacillus subtilis*) (Ye et al., 2007), but also has been demonstrated to be a promising antifungal agent and synergist with fluconazole against *C. albicans* (Huang et al., 2015; Shao et al., 2017). However, we still don’t know enough about the antifungal mechanism of SH.

Glucan and chitin are two core components of *C. albicans* inner cell wall that function as an exoskeleton and a scaffold for the external mannoproteins (McKenzie et al., 2010). In a previous study, we showed that SH alone could dramatically elevate the expressions of genes (3.71–12.63 fold) related to β-1,3-glucan synthesis and transportation including *ifd6*, *phr1*, *zap1*, *adh5*, *bgl2*, *xog1* and *fks1* (Shao et al., 2017). A former report characterized a novel resistance mechanism of *C. albicans* biofilms associated with the sequestration of fluconazole by binding to β-1,3-glucan (Nett et al., 2007). Based on these findings, we presumed that the matrix β-glucan might have physical interactions with SH and block its passage, which in turn promoted β-1,3-glucan synthesis and transportation through cell wall remodeling to unmask more glucan in response to SH attack.

In this report, the susceptibility of *C. albicans* to SH was evaluated by taking advantage of several enzymes that could lyse specific biofilm matrix chemical constituents. The interactions of SH with laminarin (commercial β-1,3-glucan) cell wall and biofilm matrix of *C. albicans* SC5314 (a widely used reference strain) and a phr1 mutant (*phr1*−/−, encoding putative β-1,3-glucanosyltransferase) were characterized by ultra-performance liquid chromatography (UPLC). The unmasking of glucan and chitin was performed by staining with monoclonal β-1,3-glucan antibody and Alexa Flour488 conjugated WGA using fluorescent microscopy.

## Materials and Methods

### Strains and Cultivation

*Candida albicans* SC5314 and *phr*−/− (Sun et al., 2015) were kindly gifted by Prof. Yuanying Jiang, School of Pharmacy, Second Military Medical University (Shanghai, China) and Prof. Guanghua Huang, Institute of Microbiology, Chinese Academy of Sciences (Beijing, China). All stock cultures of these strains were routinely maintained in sabouraud’s agar and were propagated in liquid sabouraud medium (Hope Biotech. Co., Qingdao, Shangdong, China) at 37°C for 12–16 h till the strains reached the exponential growth phase. The revived *Candida* cells were pooled with 3000 g centrifugation. After washing twice by sterile phosphate-buffered saline (PBS, Leagene, Beijing, China), the fungal cells were resuspended in RPMI-1640 medium (Invitrogen, Carlsbad, CA, United States) at pH 7.5 adjusted by 1 M NaOH.

### Susceptibility Test in Planktonic and Biofilm Cells

The fungal cell initial inoculum was adjusted to 1 × 10^3^ CFU/mL. The minimum inhibitory concentrations (MICs) of SH (Kailai Bioengineering, Xi’an, China) were performed in a 96-well flat-bottomed microplate (Corning, NA, United States) by microdilution method based on CLSI M27-A3 (CLSI, 2008). The final drug concentrations were serially two-fold diluted in a range of 1–512 μg/mL. The fungal cells were incubated with the drug used at 37°C for 48 h. The minimum inhibitory concentration (MIC) was defined as the lowest drug concentration that caused no visible cell growth. The determination of SMIC was performed according to previous reports with less modifications (Ramage et al., 2001; Pierce et al., 2008). In brief, the initial inoculum was adjusted to 1 × 10^6^ CFU/mL, and the SH concentrations were set in a range of 2–1024 μg/mL. The fungal cells were incubated with SH at 37°C for 24 h. The sessile MIC_80_ (SMIC_80_) evaluated by XTT assay was determined as the concentration that removed 90% of fungal cells compared with the drug-free control.

### Enzymatic Analysis

Seven enzymes (all from Sigma-Aldrich) were used to test their synergism with SH against *C. albicans* SC5314 and *phr*−/−. The enzyme solutions were prepared immediately prior to use. Protease K (Cat. No. P6556) was dissolved in DEPC-treated H_2_O (Beyotime Biotechnology, Jiangsu, China) containing 1% SDS (Sigma-Aldrich). Protease XIV (Cat. No. P5147) was prepared with DEPC-treated H_2_O plus 0.5% SDS. β-N-acetyl-glucosaminidase (Cat. No. A2264), β-glucuronidase (Cat. No. G7017), DNase I (Cat. No. D5025), RNase A (Cat. No. R6513), and lyticase (Cat. No. L4025) were all directly diluted with DEPC-treated H_2_O.

The initial inoculum was adjusted to 1 × 10^6^ CFU/mL. An aliquot of inoculum (100 μL) was incubated at 37°C for 24 h to allow biofilm formation in a 96-well flat-bottomed microplate. The supernatant of *Candida* biofilm was discarded and replaced by the same volume of enzyme solutions (100 μL) at the final concentration of 50 μg/mL. After 2 h of enzymatic treatment at the optimum temperature for enzyme activity, the enzyme solutions were removed and a series of two-fold diluted SH (8–1024 μg/mL for SC5314 and 2–256 μg/mL for *phr*−/−) were added for another 24 h of incubation at 37°C. An equal volume (100 μL) of RPMI-1640 medium with fungal cells was also monitored. The control consisted of 100 μL of RPMI-1640 medium with fungal cells and 50 μg/mL enzyme tested. Following incubation, biofilms were evaluated with XTT assay according to a previous description (Shao et al., 2014). The optical density (OD) value of each well was determined by a multiple function microplate reader (SpectraMax M2e, Molecular Device, Shanghai, China) at 492 nm.

### Preparation of Laminarin, Extracellular Matrix (EM), and Cell Wall

#### Laminarin

Sodium houttuyfonate plus laminarin were dissolved in RPMI-1640 and treated with 4-fold volume of methanol (Merck, Shanghai, China) to the final concentrations of 2 and 0.25 mg/mL.

#### EM

The EM preparation was performed according to previous studies with several modifications (Al-Fattani and Douglas, 2006; Zarnowski et al., 2014). Briefly, initial inoculum of 1 × 10^6^ CFU/mL was seeded to form biofilms in a 96-well flat-bottomed microplate for 24 h at 37°C. After incubation, the supernatant was aspirated gently, and the *Candida* biofilms were scraped with a cell scraper and transferred to an Eppendorf tube with the addition of 100 μL of sterile H_2_O. The mixtures containing biofilms were vortexed vigorously for 1 min (Quick Mixer SK-1, Guowang Experimental Instrument Factory, Jiangsu, China) and sonicated mildly for 20 min of 50 kHz (DSA50-GL1, Desen Ultrasonic Equipment, Fuzhou, Fujian, China) at an amplitude of 0.45–0.55 w/cm^2^ at room temperature. The biofilm-containing solution was centrifuged at 6000 g for 3 min to separate fungal cells and matrix. The matrix-containing supernatant was mixed with SH to the same final concentration (2 mg/mL) and then treated with 4-fold volume of methanol.

#### Cell Wall

The cell wall isolation was processed based on a previous study with minor modifications (Dallies et al., 1998). Briefly, the fungal cells were separated as described, pooled and washed with sterile PBS twice. *Candida* cells were disrupted in 0.5 mL of 10 mM Tris-HCl (pH 8) in the presence of 0.5 g of glass beads (425–600 μm in diameter) for four cycles of 20 s each by vortexing for 1 min with 1 min intervals on ice. The percentage of cell breakage was estimated by microscopic examination and the cycles were stopped when more than 95% of the cells were broken. The cell suspension was collected and the glass beads were washed with cold Tris-HCl. The supernatant and washings were collected and centrifuged at 3800 g for 5 min. The pellet containing disaggregated cell walls was washed several times with cold deionized H_2_O until the supernatant became clear. The cell wall-containing pellet was mixed with SH to the same final concentration (2 mg/mL) and then treated with 4-fold volume of methanol.

#### Chitin

SH plus chitin from shrink shell (Sigma, Shanghai, China) were dissolved directly in methanol to the final concentrations of 2 and 1 mg/mL. The above-mentioned methanol-treated samples were centrifuged at 3000 g for 10 min and then filtered with 0.22 μm membrane prior to injection for UPLC analysis.

### UPLC Procedures

The interactions of SH with cell wall and EM were analyzed using a ACQUITY UPLC system (Waters, Shanghai, China) equipped with a C18 UPLC BEH Amide column (1.7 μm, 3.0 × 100 mm, Waters). The mobile phase consisted of water (solvent A) and methanol (solvent B). The gradient elution procedure was set as follows: 0–2 min, 10%→25% B; 2–4 min, 25%→10% B. The column temperature was 35°C. The injection volume was 1 μL. The flow rate was 0.25 mL/min. The detection wavelength was 268 nm.

### Unmasking Assay

Prior to staining, the strains (=2 × 10^3^ CFU/mL) were treated with SH (=8 μg/mL) and caspofungin (5 × 10^−3^ μg/mL) for 24 h. The drug-free control only contained strains. The unmasking procedures were conducted as instructed before with a few adjustments (Sherrington et al., 2017). To stain surface exposed chitin, drug-treated fungal cells were incubated with 100 μg/mL Alexa Fluor 488 conjugated WGA (ThermoFisher Scienfiric) for 15 min. To stain surface exposed β-1,3-glucan, drug-treated fungal cells were blocked with 2% BSA Albumin Fraction V (Cat. No. 9048-46-8, Biofroxx, Shanghai, China) in PBS for 1 h and then incubated overnight with a monoclonal anti-β-1,3-glucan antibody (Cat. No. 400-2, Bioscience Supplies, Australia) diluted 1:300 in PBS at 4°C with gentle shaking. Followed by primary antibody treatment, cells were then washed three times with PBS and incubated with 1:100 diluted goat anti-mouse Ig G conjugated to Cy3 (Cat. No 143702A, Abbkine, Shanghai, China) for 1 h at 4°C. Cells were pooled by 3000 g centrifugation for 5 min after washing twice with sterile PBS and recorded by an inverted fluorescence microscope (Olympus IX81, Japan).

### Statistical Analysis

All experiments were performed in triplicate in three individual workdays. The results were recorded as mean ± standard deviation and calculated by SPSS 17.0 (SPSS Inc., Chicago, IL, United States). The data among groups were analyzed by one-way ANOVA with least significance difference (LSD) method, in which *p* < 0.05 was considered as statistically significant.

## Results

### Antifungal Activity of SH Was Enhanced by Lyticase

**FIGURE 1:**
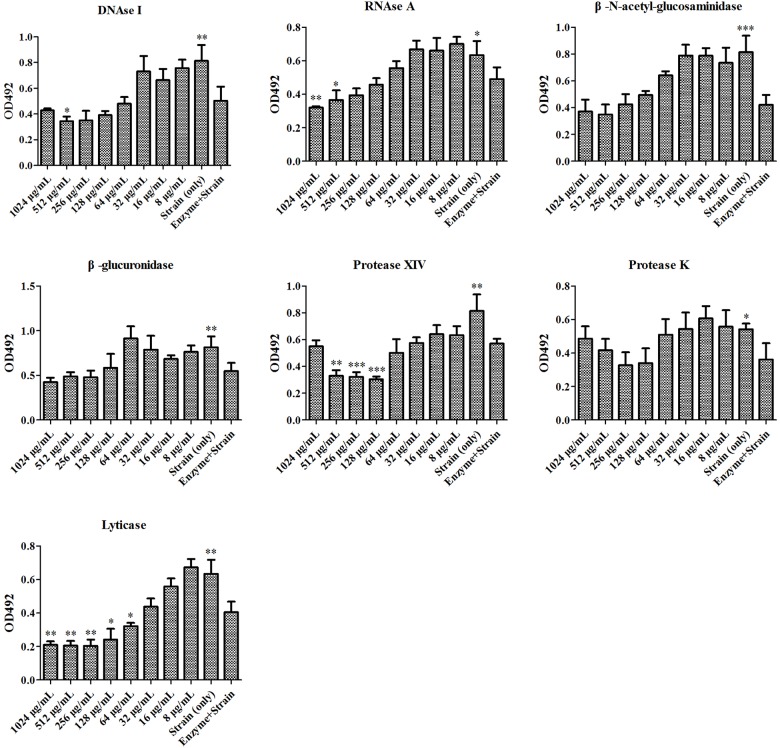
Enzymatic effects on antifungal activity of SH against *Candida albicans* SC5314. The enzymes include DNase I, RNase A, β-N-acetyl-glucosaminidase, β-glucuronidase, protease XIV, protease K, lyticase. Cells free of enzymes and drugs, enzyme-treated cells, and cells treated successively with enzymes and SH were analyzed by XTT method at 492 nm. The final concentration of enzyme was 50 μg/mL. The SH was two-fold diluted at concentrations of 8–1024 μg/mL. Other experimental procedures in Materials and Methods. ^∗^*p* < 0.05; ^∗∗^*p* < 0.01; ^∗∗∗^*p* < 0.001; compared with enzyme-treated cells.

**FIGURE 2:**
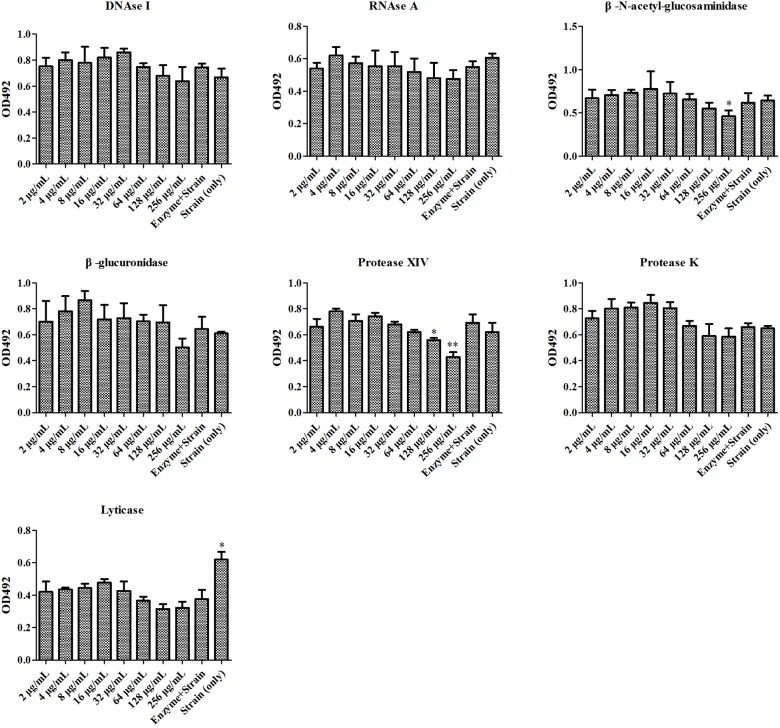
Enzymatic effects on antifungal activity of SH against phr1*–/–*. The experimental conditions were equal to Figure 1. ^∗^*p* < 0.05; ^∗∗^*p* < 0.01; compared with enzyme-treated cells.

At first, the MIC and SMIC of *phr1*−/− were determined as 32 and 64 μg/mL, respectively by microdilution method. We then used seven enzymes to hydrolyze matrix components in *C. albicans* SC5314 and *phr1*−/− biofilms. The lysis test showed that the fungal cell growth was inhibited in all of the seven enzymes alone within 2 h (Figures 1, 2), but restored to the initial concentration and viability after 24 h incubation (data not shown). The co-incubation of SH and lyticase could significantly suppress fungal proliferation at 64 μg/mL (*p* < 0.05), lower than DNAase plus SH (512 μg/mL, *p* < 0.05), RNAase plus SH (512 μg/mL, *p* < 0.05), and protease XIV plus SH (128 μg/mL, *p* < 0.001) in *C. albicans* SC5314. While the addition of SH (8–1024 μg/mL) could not promote the inhibition of the other three enzymes, i.e., β-N-acetyl-glucosaminidase, β-glucuronidase and protease K (Figure 1). In *phr1*−/−, protease XIV plus 128 or 256 μg/mL of SH (*p* < 0.05, *p* < 0.01) and β-N-acetyl-glucosaminidase plus 256 μg/mL of SH (*p* < 0.05) could generate significant inhibitions on *C. albicans* growth. The remaining five enzymes including lyticase seemed to be futile to boost SH antifungal efficacy at 2–256 μg/mL (Figure 2).

### SH Seemed to Interact With β-1,3-Glucan Physically

**FIGURE 3:**
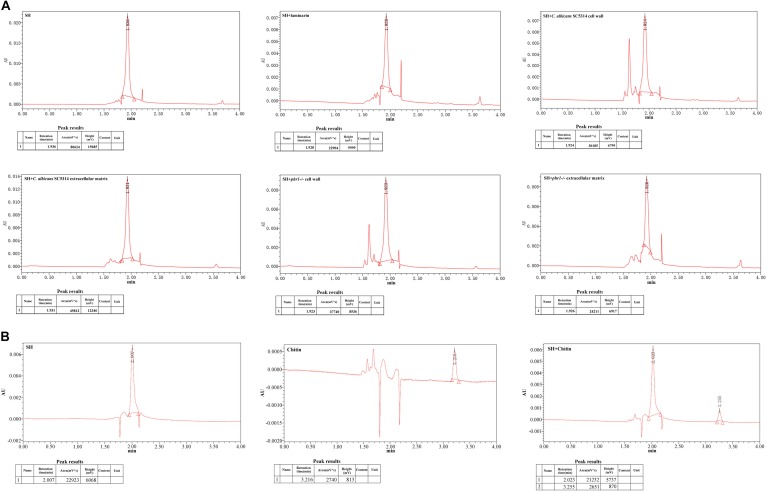
UPLC analysis under incubations of **(A)** SH, SH plus laminarin, SH plus extracted extracellular matrix of *C. albicans* 5314, SH plus disaggregated cell wall of *C. albicans* 5314, SH plus extracted extracellular matrix of *phr1–/–*, SH plus disaggregated cell wall of *phr1–/–*, and **(B)** SH, chitin, SH plus chitin. Other experimental procedures in Materials and Methods.

The enzyme lysis test indicated that the hindrance of matrix carbohydrates might be involved in the discounted antifungal effect of SH. To explore the relation of SH with matrix carbohydrates, we compared the peak areas of SH after co-incubations with laminarin (commercial β-1,3-glucan), broken fungal cell wall and disaggregated biofilm matrix by UPLC. Compared with the control (SH only), the peak area of SH markedly decreased by 71.6% after exposure to laminarin (Figure 3A). Likewise, the free SH declined greatly by 38.2 and 70% in matrix, 62.6 and 53.2% in cell wall in *C. albicans* SC5314 and *phr1*−/− (Figure 3A). In comparison, the peak area of chitin was unchanged following SH treatment (Figure 3B).

### SH Caused Cell Wall Remodeling

**FIGURE 4:**
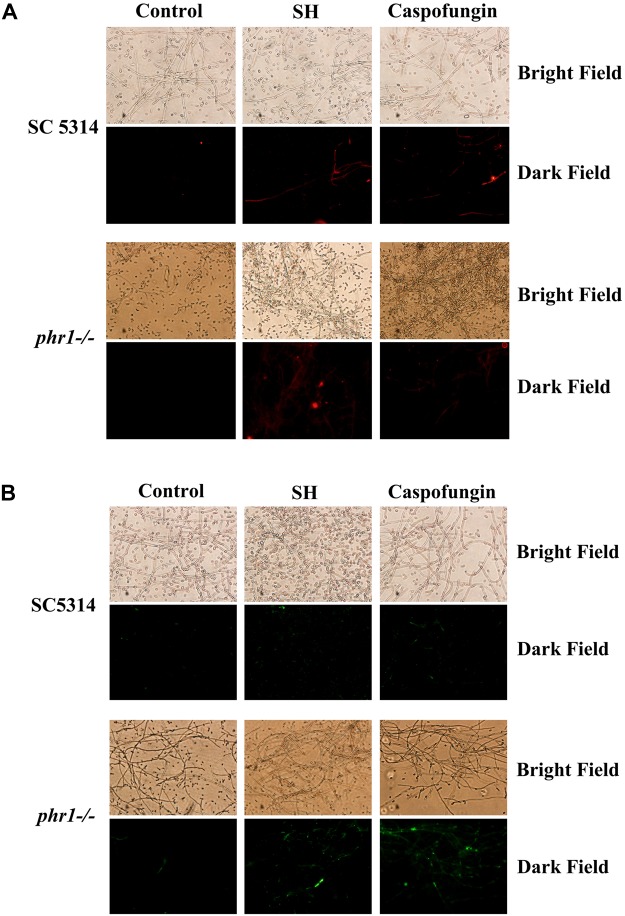
Exposure of **(A)** β-1,3-glucan and **(B)** chitin induced by SH and caspofungin through stainings with monoclonal anti-β-1,3-glucan antibody in *C. albicans* SC5314 and phr1*–/–*. Control contained drug-free fungal cells. Other experimental procedures in Materials and Methods. Magnification: ×200.

In terms of aforementioned physical interaction of SH with β-1,3-glucan, it is very likely that SH can remodel *C. albicans* cell wall and unmask β-1,3-glucan. At sub-MIC SH, the unmasking of β-1,3-glucan (red, Figure 4A) and chitin (green, Figure 4B) could be observed clearly in *C. albicans* SC5314 and *phr1*−/− compared with those without SH treatment. It seemed that β-1,3-glucan and chitin were exposed more easily in hyphae than yeasts (Figure 4).

## Discussion

### SH Disturbance Was Blocked by β-1,3-Glucan via Cell Wall Remodeling

Our previous study showed that the MIC and SMIC_90_ of SH were determined as 64 and 512 μg/mL in *C. albicans* SC5314 (Huang et al., 2015). In this work, the SMIC_90_ of SH in *phr1*−/− which had impaired biofilm matrix β-1,3-glucan delivery resulting in reduced matrix β-1,3-glucan content (Taff et al., 2012) was remarkably decreased (64 μg/mL) by eight-fold compared with that in *C. albicans* SC5314. Our hydrolase study and UPLC analysis revealed that carbohydrates (especially β-1,3-glucan) could block SH entrance into fungal cells through non-specific absorption with β-1,3-glucan (Figures 1–3). We also observed that sub-MIC SH induced β-1,3-glucan unmasking in *C. albicans* SC5314 and *phr1*−/− (Figure 4). It was reasonable to assume that β-1,3-glucan played a critical role in anti-biofilm activity of SH.

Phr1p is a putative glycosylphosphatidylinositol-anchored cell surface protein of *C. albicans* (Saporito-Irwin et al., 1995). The expression of *phr1* is regulated by pH and usually upregulated above 5.5 (Calderon et al., 2010). In *phr1* mutant cell wall of *C. albicans* and *Saccharomyces cerevisiae*, the glucan levels decreased in a diverse manner as β-1,6-glucan had a greater reduction than β-1,3-glucan (Kapteyn et al., 1997; Popolo et al., 1997; Popolo and Vai, 1998). However, it has been reported that the cell wall β-1,3-glucan levels in *phr1*−/− were comparable to those in a reference strain (SN250) (Taff et al., 2012), which was consistent with our MIC and UPLC results (Figure 3A). Intriguingly, the peak area of SH decreased markedly post-incubation with *phr1*−/− biofilm matrix which should have compromised content of β-1,3-glucan (Figure 3A). It seemed that SH was able to unmask more cell wall β-1,3-glucan in *phr1*−/− than SC5314 (Figure 4A). The increased exposure of β-1,3-glucan was parallel with previous findings that both non-functional *phr1* gene and the presence of SH triggered the upregulation of *fks1* mRNA expression (Taff et al., 2012; Shao et al., 2017). We presumed that the increased *fks1* mRNA levels did not only contribute to the accumulation of cell wall β-1,3-glucan, but could be also conductive to the transportation of β-1,3-glucan to the matrix in *phr1*−/−. In addition, *phr1* mutant changed glucan cross-linking, increased the ratio of alkali-soluble to alkali-insoluble glucan, and released β-1,3-glucan into the culture medium (Kapteyn et al., 1997; Popolo et al., 1997; Popolo and Vai, 1998; Ram et al., 1998). As a matter of fact, a considerable quantity of glucose (≈12.5%) was detected in *Candida* matrix (Zarnowski et al., 2014). Besides the above possibilities, there might be another two plausible answers to the matrix β-1,3-glucan elevation. One is that there were at least two other functional genes responsible for glucan transportation besides *phr1*, i.e., *bgl2* and *xog1* (Taff et al., 2012). The other is that the matrix-associated *zap1* pathway was independent of *phr1*, *bgl2* and *xog1* and could regulate matrix glucan levels (Nobile et al., 2009). In accordance with our findings, we hypothesized that the interaction of SH with alkali-insoluble glucan might be the main factor to block SH entrance and limit the antifungal activity of SH, while alkali-soluble glucan might be in a loose interaction with SH with less impact on SH permeation. This dynamic balance between cell wall and matrix in *phr1*−/− was in accordance with the theory that influence in the fungal cell glucan production was in the control of matrix glucan levels (Taff et al., 2012).

Considering these results, the acceptable scenario would be that *C. albicans* sensed SH attack via physical interaction with β-1,3-glucan in the cell wall, induced more β-1,3-glucan unmasking and enhanced matrix β-1,3-glucan levels to hold back further SH insults.

### SH Had a Different Antifungal Mode of Action From Fluconazole and Echinocandins

As the first contact point, the fungal cell wall is inclined to remodel in response to external stimulants (such as antifungal drug) (Hopke et al., 2018). Fluconazole has been demonstrated to be sequestrated by β-1,3-glucan in *C. albicans* biofilm matrix non-specifically but was unable to expose cell wall glucan (Wheeler et al., 2008). Caspofungin and micafungin could unmask β-1,3-glucan at sub-MIC, however, their interactions with β-1,3-glucan was unclear (Wheeler and Fink, 2006; Guirao-Abad et al., 2018). Our results displayed that SH was not only hindered by β-1,3-glucan via unknown interaction but also capable of unmasking β-1,3-glucan. Although the expressions of the glucan synthesis and delivery pathway associated genes were affected at different degrees as described, enhanced glucan exposure might be attributed to the increased synthesis and transportation of β-1,3-glucan at sub-MIC of SH which could upregulate significantly relevant gene expressions (Shao et al., 2017). In the case of caspofungin treatment, *phr2*, *kre5* and *ssn8* but not *phr1* were believed to play a major role in β-1,3-glucan unmasking (Wheeler and Fink, 2006). Conversely, we demonstrated that *phr1* was capable of promoting glucan unmasking compared with C. albicans SC5314 at sub-MIC of SH (Figure 4A) implying that *phr1* acted as a negative factor on glucan unmasking in the presence of SH.

In spite of the absence of evidence that could confirm the interaction of fluconazole and echinocandins with chitin, our results showed that chitin seemed to act as a possible physical barrier against SH without absorption (Figure 3B). Meantime, we also observed that sub-MIC of SH could cause chitin unmasking. According to previous studies, the chitin level of cell wall was increased strikingly under caspofungin treatment (Walker et al., 2008). The elevation of chitin was supposed to be a salvation to complement the decreased β-1,3-glucan caused by caspofungin in fungal cell wall to keep cell wall intact (Cormack et al., 2008; Walker et al., 2013). This caspofungin-induced salvation process was implicated with PKC, HOG, and Ca^2+^ signaling pathways (Munro et al., 2007). Nevertheless, the three pathways might play varying roles when *C. albicans* is subjected to different environmental cues including antifungal agents (Morschhäuser et al., 2016; Sherrington et al., 2017). Herein, SH unmasked considerable chitin in *phr1*−/− compared with *C. albicans* SC5314, indicating *phr1* was also involved and played a negative role in chitin exposure. It has been suggested that in *phr1*−/− cell wall of *C. albicans*, the chitin levels could increase tremendously by 5 to 10 fold. We reasoned that increased chitin exposure might be associated with relatively high levels of chitin in *phr1*−/− cell wall (Figure 4B). Therefore, the antifungal mechanism of SH was different from that of fluconazole and echinocandins.

### β-1,3-Glucan Unmasking Can Be an Alternative Parameter for Discovery of Novel Antifungal Agents

Most knowledge relevant to drug target is achieved from conventional antifungal agents, i.e., azoles, polyenes, and echinocandins. Azoles can inhibit the lanosterol 14-α-demethylase enzyme encoded by *erg11* and generate a toxic membrane sterol instead of the regular ergosterol one. Polyenes can bind and extract ergosterol from cell membrane to form membrane-spanning channels. Echinocandins can non-competitively inhibit the critical enzyme encoded by *fks1* responsible for β-1,3-glucan synthesis (Robbins et al., 2017). Coincidently, β-1,3-glucan but not ergosterol plays a structural and adhesive role in *Candida* biofilm matrix (Taff et al., 2012). Moreover, *C. albicans*-originated β-1,3-glucan could increase the resistance of *E. coli* to ofloxacin (De Brucker et al., 2015), and the β-1,3-glucan mutants would greatly improve the efficacy of antifungal agent (Nett et al., 2007; Taff et al., 2012). A list of reports proposed that the sequestration of azoles by β-1,3-glucan was a newly biofilm resistance mechanism (Nett et al., 2007, 2010a,b, 2011; Mitchell et al., 2013). Additionally, β-1,3-glucan is a safe target as it does not exist in mammalian cell membrane.

It is known that β-glucan can be recognized by dectin-1 which is a C-type lectin receptor and expressed widely on phagocytes and dendritic cells (Brown and Gordon, 2001). Mounting evidence demonstrated that their interactive recognition triggers the immunological response to β-glucan and subsequent removal of *C. albicans* (Wheeler and Fink, 2006; Galan-Diez et al., 2010; Bain et al., 2014; Morschhäuser et al., 2016; Sherrington et al., 2017; Guirao-Abad et al., 2018). A quantity of potential antifungal drugs can’t be considered in *in vitro* susceptibility test since their MICs are usually quite high (above 100 μg/mL). Whereas, the drugs take effect in internal bodies in which the host immune system should be taken into account. The β-glucan unmasking can be seen as a positive sign of activating innate immune response. In line with this understanding, we recognized that β-1,3-glucan unmasking can be an optional parameter for the discovery of novel antifungal agents.

In summary, our results showed that β-1,3-glucan could hinder the entrance of SH through non-specific absorption, resulting in unmasking β-1,3-glucan and chitin. This mode of action will be helpful for deep insights into the potential antifungal mechanism of SH and pave a way for searching novel anti-*Candida* drugs.

## Author Contributions

WD, QL, GS, and TW performed the experiments. JS and DW wrote the manuscript. JS and CW devised the program.

## Conflict of Interest Statement

The authors declare that the research was conducted in the absence of any commercial or financial relationships that could be construed as a potential conflict of interest.
